# Validation of the Prostatype® P-score for predicting prostate cancer specific mortality in a multiethnic U.S. veterans cohort

**DOI:** 10.1038/s41391-025-01070-8

**Published:** 2026-01-07

**Authors:** Alexandra Mack, Trung Duong Tran, Emelie Berglund, Gerald L. Andriole, Christopher Alley, Anthony E. Sisk, Iveth Estrada-Reyes, Kara Bissell, Haleigh Bellerose, Aubrey Jarman, Anna Hoffmeyer, Michael Burns, Sergio Sanders, Eric Vail, Andy Pao, Raja Khurram, Amal Ahmed, Stephen J. Freedland

**Affiliations:** 1 Department of Urology, Samuel Oschin Comprehensive Cancer Institute, Cedars-Sinai Medical Center, Los Angeles, CA USA; 2 Division of Urology, Veterans Affairs Health Care System, Durham, NC USA; 3 Prostatype Genomics AB, Nacka Strand, 131 52 Augustendalsvägen 20, Sweden; 4 Division of Pathology and Laboratory Medicine, University of California, Los Angeles, CA USA; 5 Department of Pathology and Laboratory Medicine, Cedars-Sinai Medical Center, Los Angeles, CA USA

**Keywords:** Predictive markers, Prostate cancer, Translational research

## Abstract

**Background:**

The Prostatype® Test evaluates expression levels of three stem cell genes (IGFBP3, F3, and VGLL3), which are combined with PSA, stage, and grade to calculate P-score. Previous research found P-score accurately predicts prostate cancer (PC) specific mortality (PCSM) in patients with newly diagnosed clinically localized PC. We evaluated the performance of P-score to predict PCSM in a large, multiethnic cohort from the Veterans’ Administration (VA).

**Methods:**

After pathologic review to ensure sufficient tumor tissue, formalin-fixed paraffin-embedded (FFPE) biopsy cores from patients with newly diagnosed PC at the Durham VA were sent to an academic medical center. There, cores were sectioned, RNA extracted, and reverse transcription quantitative polymerase chain reaction (RT-qPCR) tests conducted for IGFBP3, F3, VGLL3, and GAPDH (control). Results were combined with clinical data to generate P-scores. The association between P-score and PCSM was evaluated using c-index, Cox and Fine-Gray models, and decision curve analysis (DCA).

**Results:**

Higher P-scores were significantly associated with a higher risk of PCSM (HR = 1.48 per 1 unit increase in P-score, 95% CI: 1.20–1.84, *p* <0.001) and accurately estimated PCSM (c-index = 0.87). Adding clinical variables to P-score only incrementally improved accuracy. The DCA indicated P-score provided net clinical benefit for patients with PCSM risk between 5% and ~50%. As P-score strongly correlated with risk group, we tested the value of P-score in intermediate-risk patients specifically, where it significantly predicted PCSM (HR 1.43, 95% CI: 1.09–1.86, *p* = 0.009).

**Conclusion:**

In this American cohort of veterans, P-score significantly predicted PCSM. Adding clinical variables minimally improved accuracy. Accuracy remained high in intermediate-risk patients, wherein there is arguably the greatest need for better risk stratification. Given P-scores can be generated rapidly in-house using a standardized RT-qPCR assay, P-score represents a robust new tool to risk-stratify newly diagnosed patients for PC death, thereby minimizing mismatched treatments.

## Introduction

Despite advances in PC diagnosis and risk stratification, current methods of predicting PC aggressiveness remain suboptimal. This is mainly because methods rely on qualitative clinical assessments and human interpretation rather than validated quantitative tests that consider individual patients’ PC pathology. Accordingly, patients with PC often receive mismatched treatments, leading to undertreatment of aggressive disease or overtreatment of indolent disease. Several molecular tests were developed to address this. All existing tests require centralized reference laboratories and cannot be performed locally.

To overcome limitations of other tests, Prostatype® Test, a standardized RT-qPCR assay, was developed to evaluate the expression of stem cell genes IGFBP3, F3, and VGLL3 (the three-gene signature). Prostatype® Test can be performed locally either in hospital molecular laboratories (Europe) or designated CLIA-certified facilities (U.S.) (i.e., “in-house”), allowing faster and more flexible implementation.

The three-gene signature significantly predicted overall and PC-specific mortality (PCSM) in a Swedish cohort of 189 PC patients diagnosed between 1986 and 2001 [1]. Another study evaluated whether adding the expression levels of IGFBP3 and F3 from formalin-fixed paraffin-embedded (FFPE) prostate biopsies could improve the prediction of overall survival compared to clinical parameters alone in 241 PC patients. The results showed that combining the three-gene signature with PSA, Gleason score, and tumor stage at diagnosis significantly improved survival prediction accuracy [2]. A follow-up study developed the integrated Prostatype score (P-score) by combining the three-gene signature with serum PSA, Gleason score, and clinical T-stage. The study demonstrated that continuous P-score (0–15) could be categorized into low- (0–2), intermediate- (3–5), and high-risk (6–15) groups, which significantly predicted PCSM [3]. The categorized P-score has since been locked and validated in independent cohorts from Taiwan, Spain, and Sweden [4–6]. Whether P-score predicts outcomes in more diverse populations outside of Europe and Asia remains untested.

To assess the performance of P-scores in an American cohort, we analyzed a multiethnic cohort of PC patients from a Veterans Administration (VA) hospital and conducted assays in-house at an academic center, correlating resultant P-scores with PCSM among patients newly diagnosed with clinically localized PC. We hypothesized P-scores would significantly and accurately predict PCSM, providing unique information above and beyond standard clinical variables.

## Methods

### Study design and participants

After obtaining approval with waivers of written consent from the Durham VA IRB, we identified patients diagnosed January 1, 2002, to December 31, 2019, at the Durham VA with very low- to high-risk PC [7] with diagnostic biopsy tissue available. Patients were excluded if they had a history of cancers other than PC (excluding basal cell or squamous cell skin cancers) prior to PC diagnosis, were diagnosed with very high-risk PC, or had clinical evidence of metastasis at diagnosis. After reviewing charts and confirming tissue availability, 1531 patients were eligible (Supplementary Fig. 1).

Patients’ biopsy tissue blocks and their corresponding hematoxylin and eosin slides were reviewed by an expert pathologist. 729 patients were excluded after pathology review due to a lack of or limited tissue. FFPE tissue blocks from the remaining 802 unique patients were sent to Cedars-Sinai Medical Center for further evaluation. Four to ten sections (dependent on tissue availability) at ≥8 µm thickness were sectioned from the FFPE blocks and reviewed by a molecular pathologist to select samples with ≥50% tumor content. This step resulted in the exclusion of 452 patients. Sample insufficiency is attributed to increased tissue requirements of pre-2012 PC diagnosis methods and outdated, unstable FFPE materials from that time.

From the remaining 349 patients, RNA was extracted and underwent RT-qPCR assay for the three-gene signature, as well as GAPDH. P-scores were calculated using a locked and validated algorithm that integrates gene expression of VGLL3, IGFBP3, and F3 (measured as ΔCT values normalized to GAPDH) together with clinical variables (PSA, Gleason score, and clinical T-stage). The resulting score (the exact algorithm is proprietary) ranges from 0 to 15, with higher values indicating increased risk [3]. Of the 349 patients tested, more samples from 2013 to 2019 met the GADPH threshold for Prostatype Testing compared to samples from 2002 to 2012 (Supplementary Table 1), and samples from 2018-2019 outperformed all others. Given that a prior study found that the choice of biopsy core did not impact the prognostic performance of P-scores [8], and a more recent validation confirmed consistent test results across tumor foci [9], we included two tissue samples for some patients (*N* = 6). P-scores for these 6 patients matched exactly (*N* = 2), differed by 1 point (*N* = 2), and differed by 2 points (*N* = 2). Importantly, none of these differences placed patients in higher (or lower) P-score risk groups. When more than one P-score was available, we used the higher score. In total, 160 patient samples met P-score threshold for GAPDH for ΔCT values (CT values < 28) and were included in the final study population.

### Statistical analysis

Descriptive statistics were generated for patient characteristics (Table 1) with medians (IQR) for continuous variables (age, BMI, prostate and PSA characteristics, and follow-up time); frequencies and percentages for categorical variables (biopsy characteristics, grade group, primary therapy type, NCCN risk group and cause of death); and P-scores divided into a priori defined low- (0–2), intermediate- (3–5) and high-risk (6–15) groups and stratified.

**Table 1:** Cohort demographic and clinical characteristics by P-score.

	All patients (*N *= 160)	P-score			
		Low risk (0–2) (*N* = 22)	Intermediate risk (3–5) (*N* = 75)	High risk (6–15) (*N* = 63)	*P* value
**Age at diagnosis (years)**					0.069^a^
Median (IQR)	65 (60, 69)	62 (58, 67)	65 (60, 70)	65 (60, 70)	
**Race,** ***n*** **(%)**					0.710^b^
Black	116 (73%)	17 (77%)	51 (68%)	48 (76%)	
White	43 (27%)	5 (23%)	23 (31%)	15 (24%)	
American Indian or Alaska Native	1 (0.6%)	0 (0.0%)	1 (1%)	0 (0.0%)	
**BMI (kg/m**^**2**^**)**					0.542^a^
Median (IQR)	28.7 (25.3, 33.0)	28.4 (25.4, 32.3)	29.9 (25.5, 33.2)	27.8 (24.1, 33.0)	
**Number of positive cores**					<0.001^a^
Median (IQR)	6.0 (4.0, 8.0)	4.0 (3.0, 6.0)	5.0 (3.0, 7.0)	7.0 (5.0, 9.0)	
**Clinical stage**					0.004^b^
T1c	89 (56%)	13 (59%)	52 (69%)	24 (38%)	
T2a	41 (26%)	7 (32%)	15 (20%)	19 (30%)	
T2b	14 (8.8%)	0 (0.0%)	3 (4.0%)	11 (18%)	
T2c	16 (10%)	2 (9.1%)	5 (6.7%)	9 (14.3%)	
**Grade group,** ***n*** **(%)**					<0.001^b^
1	35 (22%)	15 (68%)	19 (25%)	1 (1.6%)	
2	57 (36%)	6 (27%)	31 (41%)	20 (32%)	
3	46 (29%)	1 (4.5%)	21 (28%)	24 (38%)	
4	12 (7.5%)	0 (0.0%)	4 (5%)	8 (13%)	
5	10 (6.3%)	0 (0.0%)	0 (0.0%)	10 (16%)	
**PSA (ng/ml), pre-biopsy**					<0.001^a^
Median (IQR)	7.7 (5.4, 14.8)	5.1 (4.7, 5.5)	6.7 (5.0, 8.7)	14.9 (9.3, 23.1)	
**Prostate volume from ultrasound or MRI (mL)**					0.206^a^
Missing	5	1	1	3	
Median (IQR)	33.0 (24.3, 45.0)	25 (20, 43)	35 (24, 43.0)	33 (26, 49)	
**Primary therapy type**					<0.001^b^
Unknown	1 (0.6%)	0 (0.0%)	0 (0.0%)	1 (1.6%)	
RP	60 (38%)	8 (36%)	37 (49%)	15 (24%)	
Cryotherapy	2 (1.3%)	0 (0.0%)	1 (1.3%)	1 (1.6%)	
Hormonal	15 (9.4%)	0 (0.0%)	2 (2.7%)	13 (21%)	
No treatment/ Active surveillance	14 (8.8%)	5 (23%)	3 (4.0%)	6 (9.5%)	
Radiation ± ADT	68 (43%)	9 (41%)	32 (43%)	27 (43%)	
**NCCN risk group,** ***n*** **(%)**					<0.001^b^
Very Low/Low	28 (18%)	13 (59%)	15 (20%)	0 (0.0%)	
Favorable Intermediate	21 (13%)	2 (9%)	15 (20%)	4 (6.3%)	
Unfavorable Intermediate	71 (44%)	7 (32%)	40 (53%)	24 (38%)	
High	40 (25%)	0 (0.0%)	5 (6.7%)	35 (56%)	
**Cause of death,** ***n*** **(%)**					0.001^b^
Alive	107 (67%)	20 (91%)	57 (76.0%)	30 (48%)	
Die of prostate cancer	14 (8.8%)	0 (0.0%)	4 (5.3%)	10 (16%)	
Die of non-prostate cancer causes	37 (23%)	2 (9.1%)	14 (19%)	21 (33%)	
Unknown	2 (1.3%)	0 (0.0%)	0 (0.0%)	2 (3.2%)	
**Follow-up time for prostate cancer mortality (years)**					0.363^a^
Median (IQR)	7.5 (5.5, 10.7)	7.7 (6.2, 9.7)	7.7 (5.8, 10.7)	7.2 (3.5, 11.2)	

^a^Kruskal–Wallis *p* value.

^b^Fisher Exact *p* value.

We evaluated differences in characteristics among risk groups using Kruskal–Wallis tests for continuous variables. Fisher’s exact test assessed any association between race, cancer characteristics, and cause of death among risk groups.

As competing risks were present in our analysis of PCSM (i.e., death from non-PC causes), we modeled both cause-specific hazard and sub-distribution hazard under the Fine-Gray method. As these two models can yield different results, prior work recommended presenting both [10].

Cumulative incidence functions were estimated and stratified by pre-defined P-score groups: low (0–2), intermediate (3–5), and high (6–15). Univariable cause-specific Cox and Fine-Gray models were fitted using P-score as a continuous variable to predict PCSM. Due to a few PC-specific deaths, we could not perform a full multivariable model without overfitting. Thus, we tested whether P-score provided information independent from its association with PCSM after adjusting for PSA, grade, and NCCN risk group, each in separate models.

We determined the accuracy of P-scores to predict death using concordance indices (c-index), and Areas Under the Curve (AUCs) to predict death at 10 years. The c-index over the follow-up period was based on the cause-specific Cox model assessing PCSM. The model generated risk scores for each unique patient, and the c-index was calculated by comparing all possible pairs to determine how well the model ranked those at higher risk of PCSM. To assess the clinical benefit of P-scores in predicting PCSM at 10 years, we used a decision curve analysis (DCA).

## Results

### Baseline characteristics

One hundred sixty patients, for whom demographic and clinical characteristics by P-score may be found in Table 1, were included in the cohort, with a median age of 64.5 years and a median PSA at diagnosis of 7.7 ng/mL. In this cohort, most patients were Black (73%) and the rest were White (27%) or American Indian/Native Alaskan (<1%). Most patients underwent radical prostatectomy (RP) (38%) or radiation therapy (43%).

P-scores ranged from 0 to 15, with a median of 5. When stratified by pre-defined thresholds, patients with high P-scores tended to have signs of more aggressive disease including more positive cores (*p* <0.001), higher clinical stage (*p* <0.004), higher grade group (*p* <0.001), higher PSA (*p* <0.001), higher NCCN risk group (*p* <0.001), and were more likely to have died from PC (*p* <0.001). Reflecting their higher-risk profile, patients with elevated P-scores were more frequently treated with hormonal therapy alone and were less likely to undergo RP (*p* <0.001). Notably, 6 patients (9.5%) with high-risk P-scores were managed with either no treatment or active surveillance. Several of these patients experienced poor outcomes, consistent with undertreatment relative to their high molecular risk profile.

### P-score and PC death

During a median follow-up of 7.5 years, 51 patients died—14 from PC and 37 from causes other than PC.

**Fig. 1:**
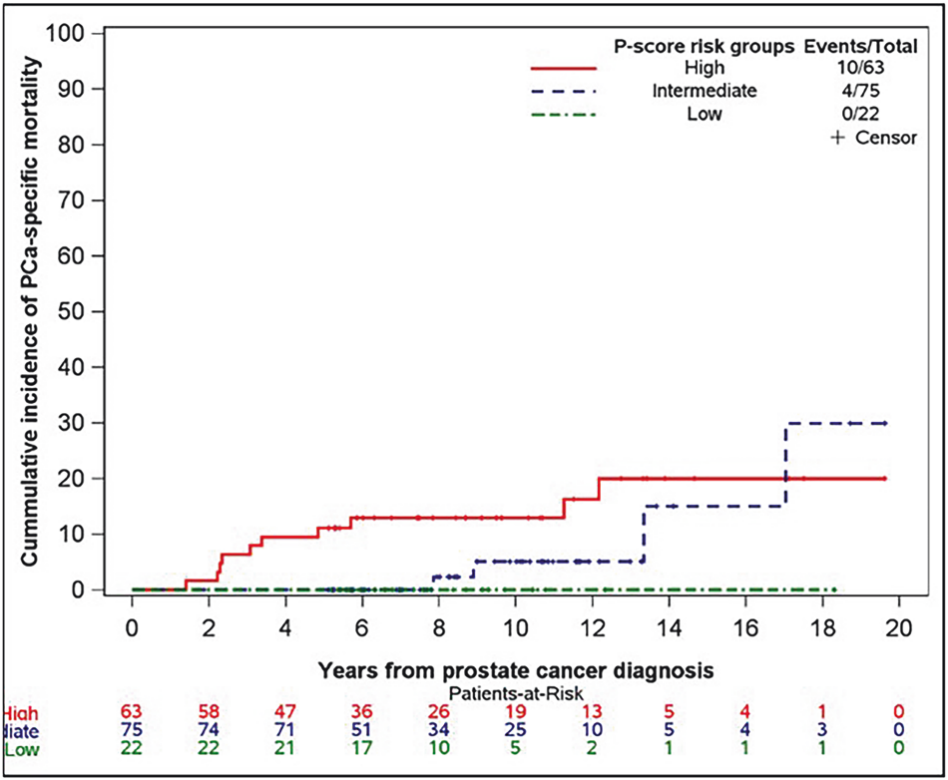
Cumulative incidence curves for PC-specific mortality from cause-specific Cox model in three P-score risk groups.

When modeled as a continuous variable, higher P-scores were significantly associated with increased risk of PCSM (HR = 1.48 per unit increase, 95% CI: 1.20–1.84; *p* <0.001; Table 2). P-score significantly separated groups based on PCSM risk (Fig. 1).

**Table 2:** Univariable and multivariable analysis of P-score predicting PCSM.

	Multivariable cause-specific Cox regression model		Multivariable Fine-Gray competing risks model	
Variables	HR (95% CI)	*P* value	HR (95% CI)	*P* value
P-score (univariable)	1.48 (1.20–1.84)	<0.001	1.33 (1.18–1.49)	<0.001
P-score + Grade	1.27 (0.97–1.67)	0.088	1.38 (1.18–1.62)	<0.001
P-score + PSA at diagnosis	1.41 (1.08–1.84)	0.012	1.19 (1.02–1.39)	0.024
P-score + NCCN risk group	1.25 (0.94–1.70)	0.131	1.33 (1.13–1.56)	0.001

Due to a few PC deaths, we were limited from performing a full multivariable model. Thus, to test whether P-score provided independent information, we adjusted for PSA, grade, and NCCN risk group in separate models (Table 2). In subsequent Fine-Gray competing risk models, P-score remained a significant predictor of PCSM regardless of clinical variable adjusted for, with HRs per 1 unit of P-score ranging from 1.19 to 1.38 (all *p* ≤ 0.024).

### Accuracy for predicting PC death

Given P-score significantly predicted PCSM after adjusting for key clinical variables, we next asked how accurately it could assess PCSM risk using both c-index (0.87) and AUC (0.80) predicting PCSM within 10 years after diagnosis (Table 3).

**Table 3:** C-Index over the entire follow-up period and AUC at 10-year follow-up from prostate cancer diagnosis for univariable and multivariable models predicting PCSM.

Model	Variable	C-index (95% CI) across follow-up	AUC (95% CI) for death at 10 years
**Univariable**			
	P-score	0.87 (0.78–0.96)	0.80 (0.61–0.99)
	Grade	0.82 (0.72–0.92)	0.74 (0.53–0.95)
	PSA at diagnosis	0.74 (0.57–0.91)	0.69 (0.47–0.91)
	NCCN risk group	0.83 (0.75–0.91)	0.75 (0.57–0.93)
**Multivariable**			
	P-score + PSA at diagnosis	0.86 (0.76–0.96)	0.80 (0.6–1.00)
	P-score + grade	0.89 (0.80–0.98)	0.80 (0.59–1.00)
	P-score + NCCN risk group	0.89 (0.80–0.98)	0.80 (0.59–1.00)

P-score demonstrated high accuracy for predicting PCSM as well as 10-year PCSM, outperforming PSA and grade individually. Although the NCCN risk group, which incorporates PSA, grade, and clinical stage, showed the highest accuracy among models using clinical variables, it remained inferior to P-score. Adding clinical variables to P-score resulted in only marginal improvements in predictive performance.

### Decision curve analysis

**Fig. 2:**
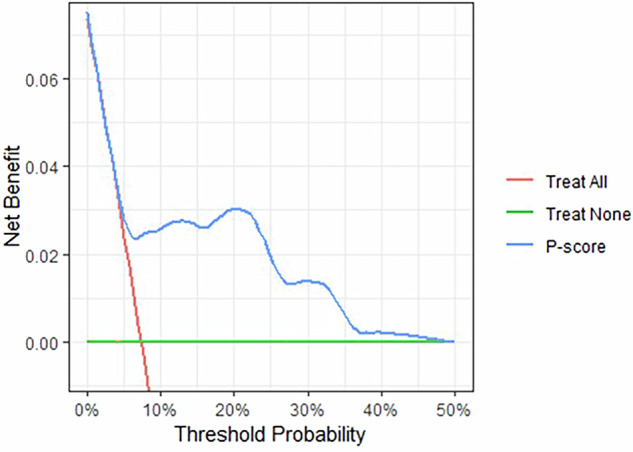
Decision Curve Analysis (DCA) evaluating the clinical benefit of predicted risk for prostate cancer-specific death at 10 years.

To evaluate the clinical benefit of P-score, we performed a DCA (Fig. 2). The analysis showed that using P-score to guide clinical decision-making yielded positive net benefit across a wide range of threshold probabilities for PCSM, ranging from 5 to ~50%.

### Sensitivity analysis

Given the lack of PC deaths in the low-risk group and that P-score strongly correlated with the risk group, we tested the value of P-score in intermediate-risk patients, the group that would arguably benefit most from further risk stratification. Even in this more restrictive population, P-score remained a significant predictor of PCSM on Fine-Gray analysis with very similar hazard ratios to the full cohort (HR 1.43, 95% CI: 1.09–1.86, p = 0.009).

## Discussion

Risk stratification of clinically localized PC remains suboptimal. Quick, easily obtained biomarkers are needed to improve predictions of PC aggressiveness. We evaluated P-score to understand its capability in predicting PCSM in our multiethnic cohort of PC patients. We found P-score was a significant predictor of PCSM with high accuracy. Adjustment for or addition of standard clinical variables had a limited incremental impact on the predictive accuracy, highlighting P-score’s potential value as a robust prognostic tool. Importantly, it provided accurate risk assessment even in intermediate-risk patients. These data support that P-score is an accurate and valid predictor of PCSM among patients with clinically localized PC.

Historically, PC risk stratification was based on PSA, stage, and grade [11]. More recently, gene expression levels within the tumor have gained interest. There are 3 commercially available tests using tumor gene expression to aid risk stratification [12]. While all three tests perform well, they are sent out to labs, which increases wait times for results. As such, an unmet need in the field is a fast, flexible, in-house assay that accurately predicts PCSM.

In our cohort of newly diagnosed PC patients, we found P-score significantly predicted PCSM with high accuracy (c-index = 0.87). Prior studies have also shown high accuracy in newly diagnosed PC patients, including in a cohort of 316 in Sweden (AUC = 0.93) [6], a cohort of 93 in Spain (AUC = 0.81) [5], and a cohort of 92 in Taiwan (c-index = 0.90) [4]. These findings validate P-score performance across diverse healthcare settings and patient populations. These studies, together with ours, suggest a performance of P-score ranging from 0.80 to 0.93. Notably, this level of accuracy for predicting PCSM compares well to other commercially available tests for PC risk stratification, such as Decipher (c-index=0.85) [13], Prolaris (c-index=0.78 for 10-year PCSM) [14], and Oncotype (time-dependent AUC = 0.84) [15]. Our current results, along with those from prior studies, support that P-score, easily generated in-house, can accurately predict PCSM and may be a valuable tool for PC risk stratification.

When developing new biomarkers, it is crucial to ask whether they provide information above and beyond what can be obtained by standard clinical variables. It is notable that P-score outperformed PSA, grade, or risk group, which combines PSA, stage, and grade. P-score’s superior performance compared to these factors alone suggests that the three-gene signature adds unique prognostic value. This is reflected in Table 3, where P-score achieved higher accuracy than PSA or grade individually, and in multivariable Fine-Gray models, where P-score remained a significant predictor of PCSM even after adjustment for these clinical variables. Moreover, the addition of clinical variables to P-score resulted in minimal improvement in accuracy. Within the limitations of the small sample size and few PC deaths, P-score provided unique information above and beyond standard clinical variables. Indeed, P-score provided net clinical benefit beyond treating all or no patients within a PCSM range of ~5–50%. This range reflects common clinical decision thresholds when considering initiation or escalation of treatment in patients with localized PC, suggesting P-score could potentially help avoid overtreatment in low-risk individuals and undertreatment in those at higher risk. Importantly, we found a subset of patients with high P-scores managed conservatively, with either no treatment or active surveillance. These patients experienced adverse outcomes, suggesting that clinical parameters may have underestimated the biological aggressiveness of their disease. This highlights a potential clinical application of P-score—identification of patients at risk for undertreatment, thereby improving decision-making for patients with high-risk molecular profiles.

Notably, intermediate-risk PC presents a great challenge in accurate risk stratification. An important finding from our study was that P-score remained a significant predictor of PCSM among intermediate-risk patients, with accuracy on par with the full cohort. While further confirmation in larger cohorts is needed, these data support the use of P-score across the full spectrum of patients, most notably among intermediate-risk patients, where there is a great unmet need.

One key strength of P-scores is that they can be generated rapidly in-house in hospital molecular laboratories (Europe) or in designated CLIA-certified facilities (U.S.). As such, they are differentiated from other commercially available test results, which require the use of centralized reference labs. Intuitively, this should lead to lower costs. Indeed, a prior Swedish study found that use of P-scores was not only associated with improved quality-adjusted life years but also *lowered* costs [16]. Though this would need confirmation in other healthcare systems, including the US, it is noteworthy that a test might be cost-effective *and* lower healthcare costs.

An important strength of this study is that most PC patients were African American (73%), a group historically underrepresented in genomic validation studies. This complements prior studies in European and Asian populations and enhances the relevance of our findings for addressing PC disparities. We also tested whether P-score provided information above and beyond standard clinical variables and assessed its potential specifically among intermediate-risk patients. Finally, we measured PCSM rather than intermediate endpoints, which are not as well-linked with PCSM.

These strengths notwithstanding, our study had some limitations. The number of included patients was modest, and the number of PC deaths was low. This limited our power to test other PC endpoints and for a more robust multivariable adjustment. These low numbers reflect a high drop-out rate due to small amounts of tissue available in prostate biopsies. Prior to ~2016, practice patterns at the Durham VA were to include all cores from a single site (i.e., left vs. right) into one block and use a greater number of sections to make diagnoses, leaving less residual tissue for research. As such, it was sometimes challenging to create sections that contained sufficient tumor for analysis. Also, some patients in this study were included in prior research studies, and there was not sufficient tumor tissue remaining to generate P-scores. Likewise, obtaining RT-qPCR-quality RNA on 20+ year old samples was limiting. Newly acquired cores in separate jars and undegraded RNA (i.e., samples from 2018 to 2019) had higher yields for use in calculating P-scores (Supplementary Table 1). Finally, patients in this study received heterogeneous treatments, potentially impacting PCSM risk. However, we were not powered to test this or stratify patients by treatment received. As such, further testing in larger cohorts is warranted.

In a multiethnic cohort of PC patients from a VA hospital, Prostatype P-scores, derived using standardized in-house RT-qPCR assays, accurately predicted PCSM beyond standard clinical information and performed nearly equally among intermediate-risk patients as all patients. These findings support the integration of P-scores into clinical workflows for quick, accurate risk stratification of newly diagnosed PC patients, particularly among those with intermediate-risk disease.

## Supplementary information

Supplemental Figure 1

Supplemental Table 1
