# Supplementary material for: Validation of the Prostatype® P-score for predicting prostate cancer specific mortality in a multiethnic U.S. veterans cohort
Source: Prostate Cancer Prostatic Dis. 2026 Jan 7;29(3):611–6. doi: 10.1038/s41391-025-01070-8 (PMC13472856; doi:10.1038/s41391-025-01070-8)
Supplement: Supplementary file 1 [file 41391_2025_1070_MOESM2_ESM.pdf]

Biopsy Year	# Unique Patient Samples	Usable Patient Samples (%)	Passed P-Test GAPDH threshold (%)
2002-2012	553	208 (38)	74 (36)
2013-2017	178	94 (52)	70 (74)
2018-2019	71	47 (66)	44 (94)

Supplemental Table 1. Usability and P-score success on FFPE core biopsy samples by acquisition year.
